# Supplementary material for: Accelerating First-Principles Molecular-Dynamics Thermal Conductivity Calculations for Complex Systems
Source: J Chem Theory Comput. 2025 Dec 19;22(1):513–27. doi: 10.1021/acs.jctc.5c01525 (PMC12805575; doi:10.1021/acs.jctc.5c01525)
Supplement: Supplementary file 1 [file ct5c01525_si_001.pdf]

Supplementary Information for Publication Accelerating first-principles molecular-dynamics thermal conductivity calculations for complex systems

Sandro Wieser,† Yu-Jie Cen,† Georg K. H. Madsen,† and Jesús Carrete*,‡

†*Institute of Materials Chemistry, TU Wien, A-1060 Vienna, Austria*

‡*Instituto de Nanociencia y Materiales de Aragón, CSIC-Universidad de Zaragoza, E-50009 Zaragoza, Spain*

E-mail: jcarrete@unizar.es

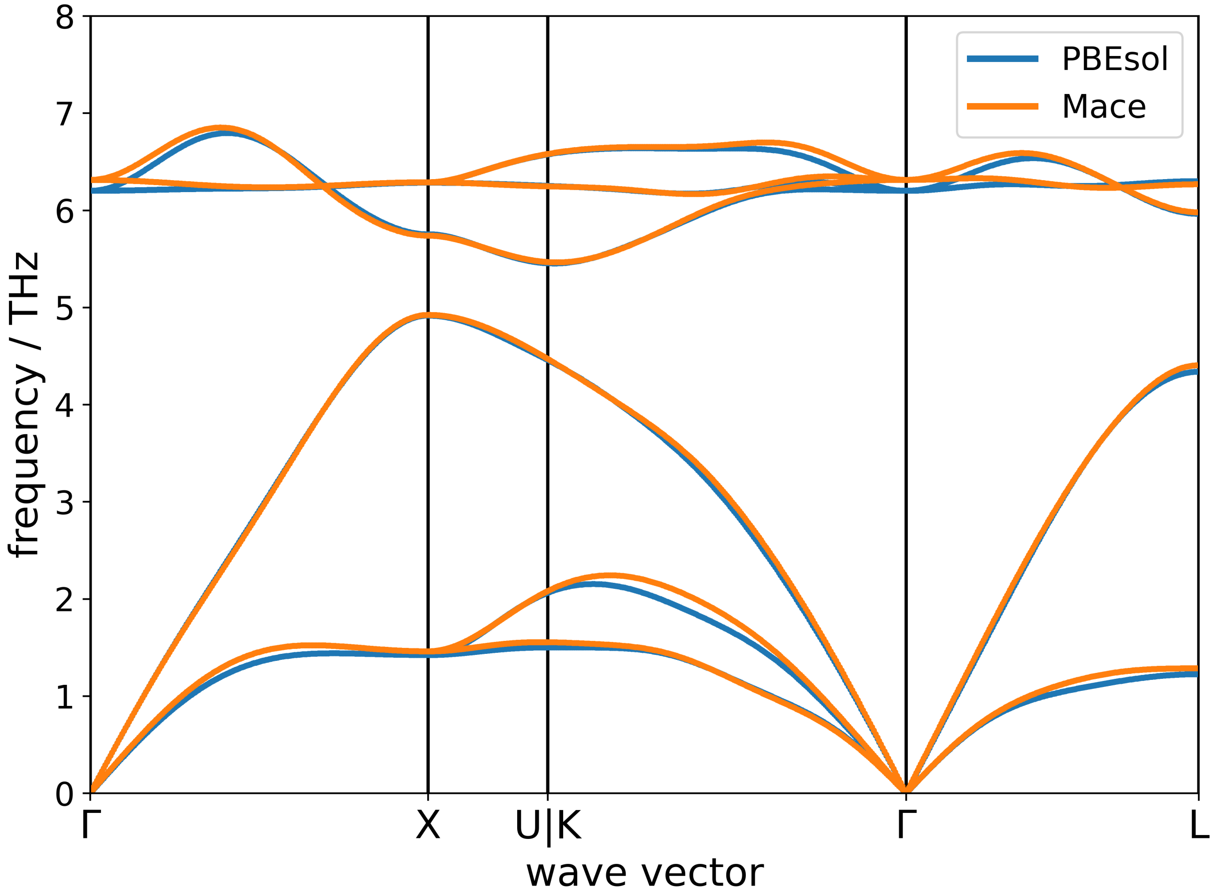

Figure S1: Phonon band structure of the InAs ZB bulk phase computed either with DFT (PBEsol) or using the trained MACE model.

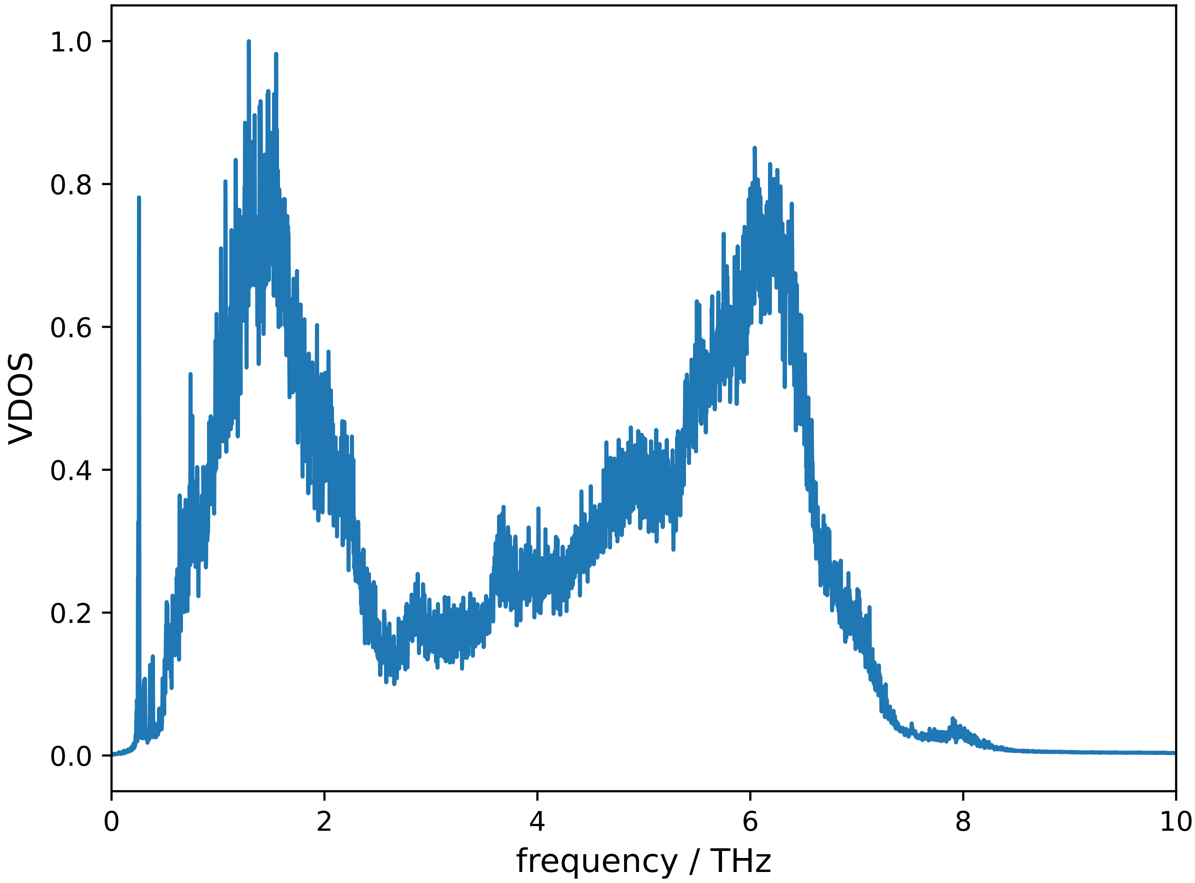

Figure S2: Vibrational density of states as obtained from the velocity autocorrelation function from the first 100 ps of a molecular dynamics simulation for the zincblende nanowire.

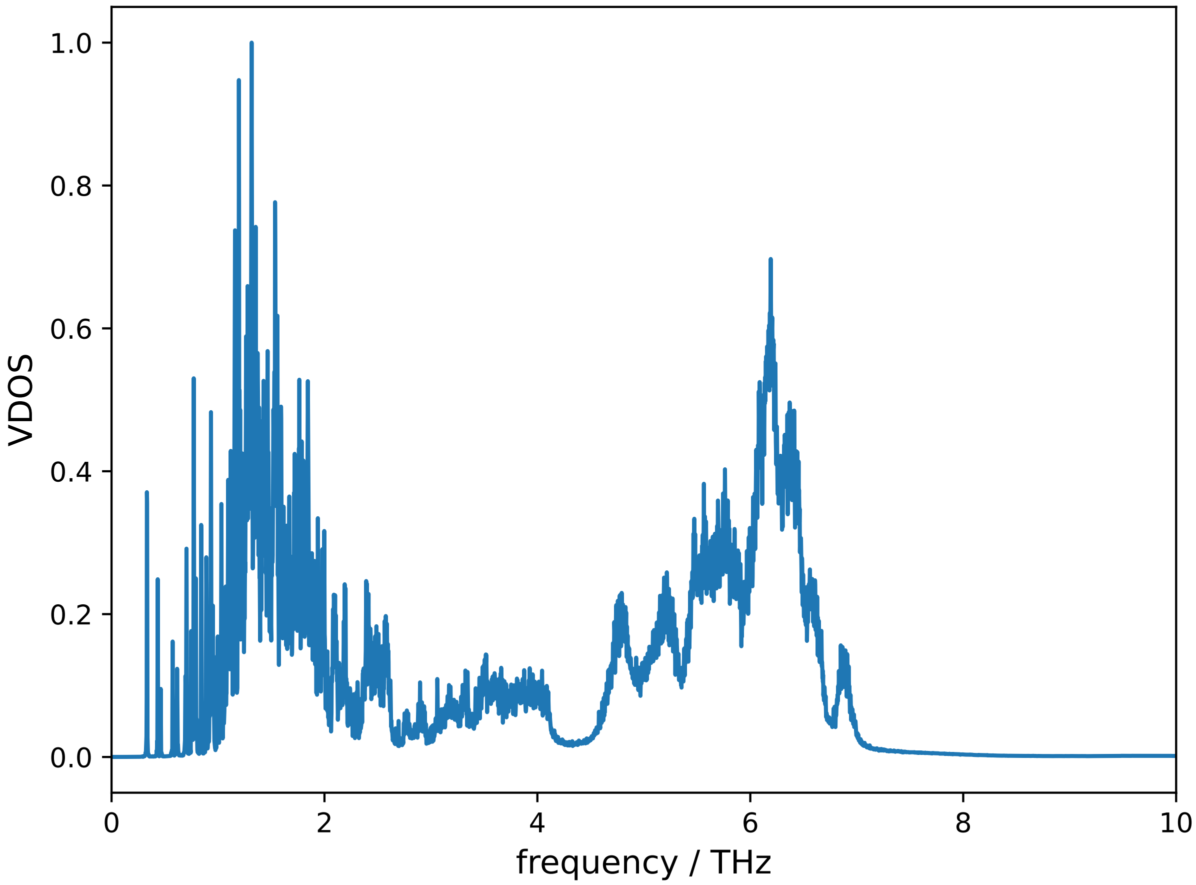

Figure S3: Vibrational density of states as obtained from the velocity autocorrelation function from the first 100 ps of a molecular dynamics simulation for the wurtzite nanowire.

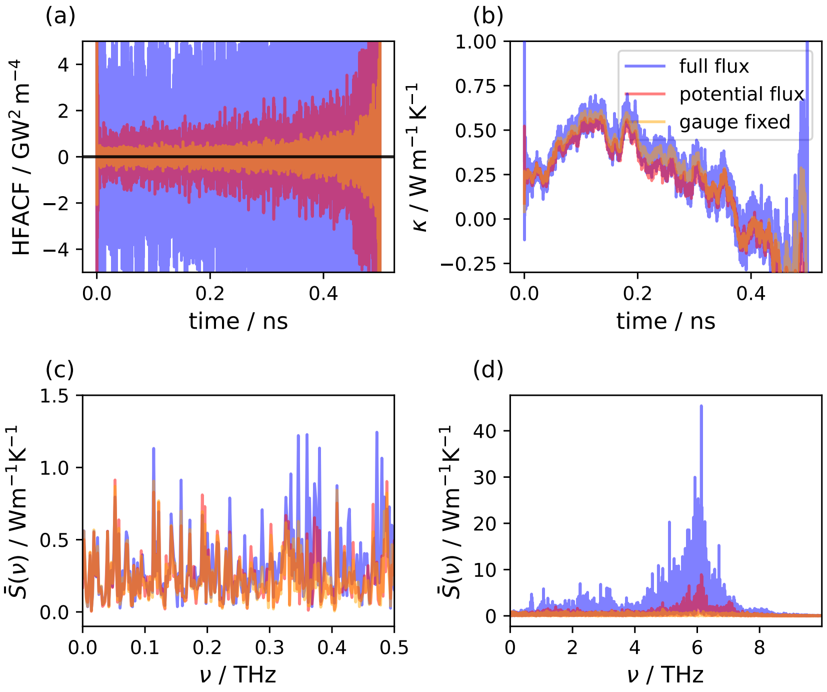

Figure S4: Comparison of different noise reduction techniques for the independent simulation number 1 for the ZB nanowire: using the full flux, removing the convective term leaving only the potential flux, and exploiting the gauge invariance of the flux. Comparisons shown are of (a) the heat flux autocorrelation functions, (b) the thermal conductivity κ, (c) the low-frequency power spectrum, and (d) the full power spectrum.

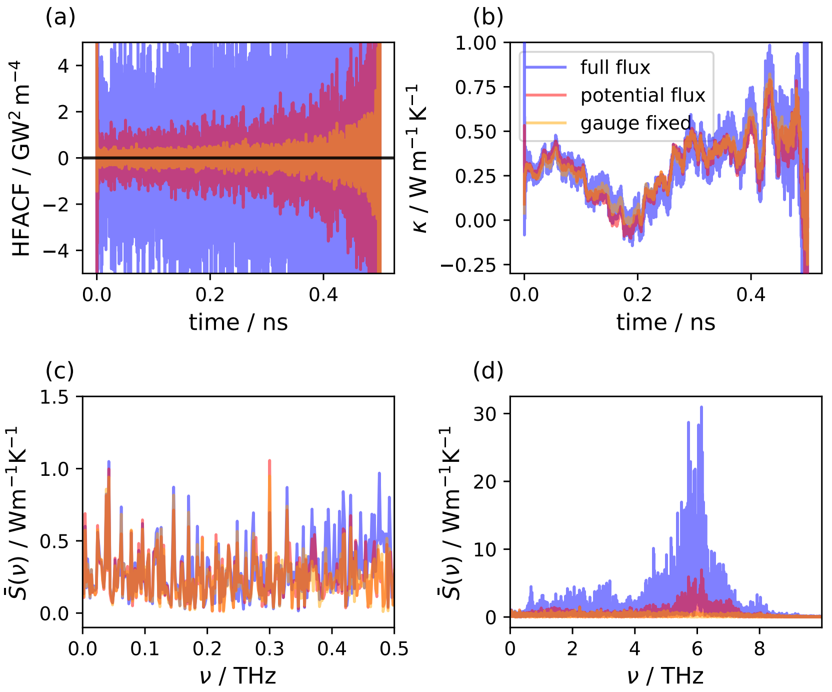

Figure S5: Comparison of different noise reduction techniques for the independent simulation number 2 for the ZB nanowire in the same style as figure S4.

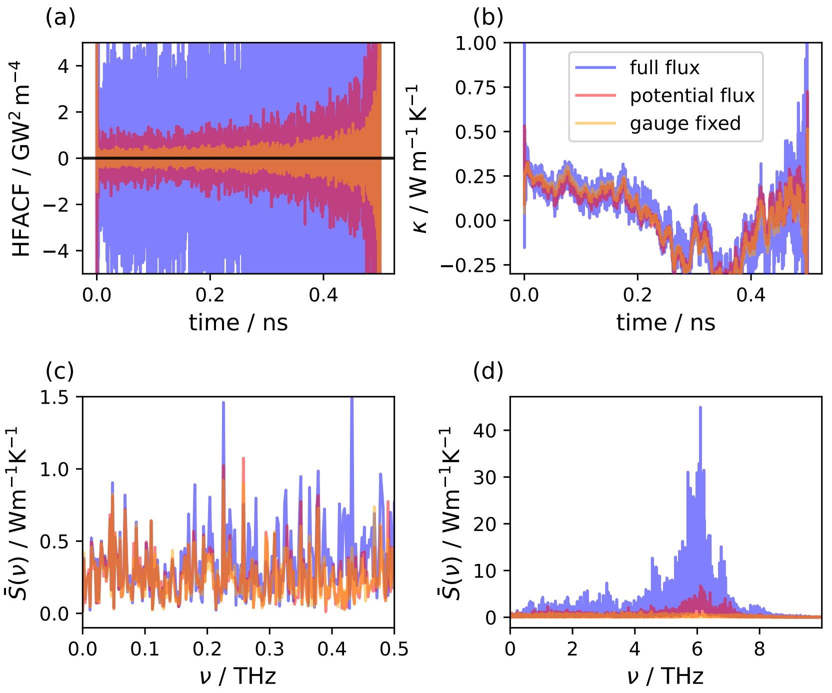

Figure S6: Comparison of different noise reduction techniques for the independent simulation number 3 for the ZB nanowire in the same style as figure S4.

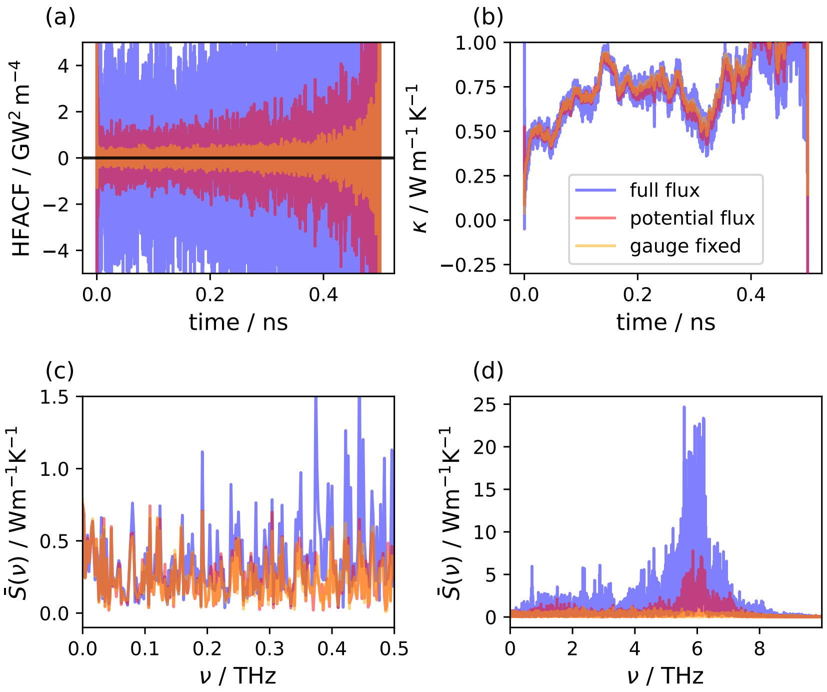

Figure S7: Comparison of different noise reduction techniques for the independent simulation number 4 for the ZB nanowire in the same style as figure S4.

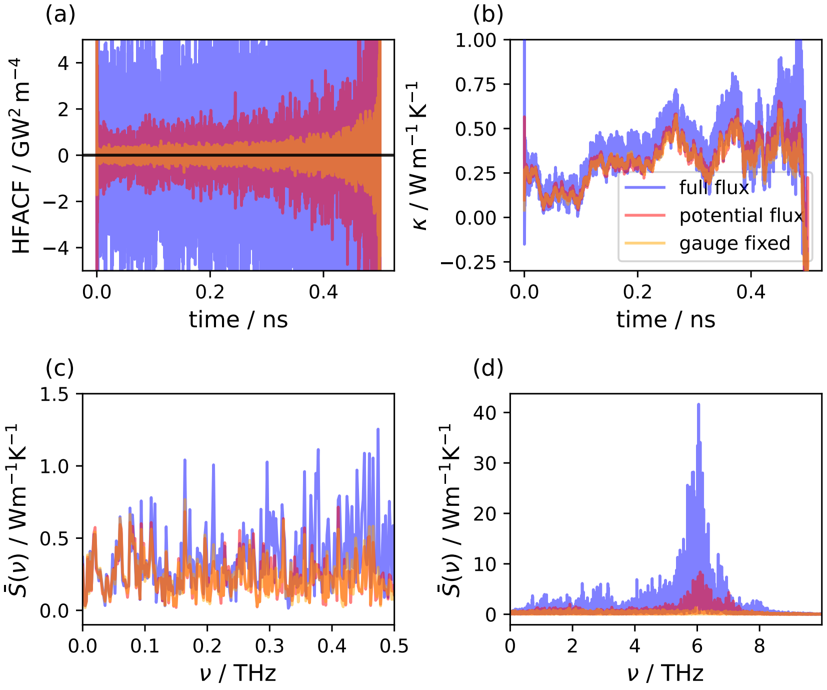

Figure S8: Comparison of different noise reduction techniques for the independent simulation number 5 for the ZB nanowire in the same style as figure S4.

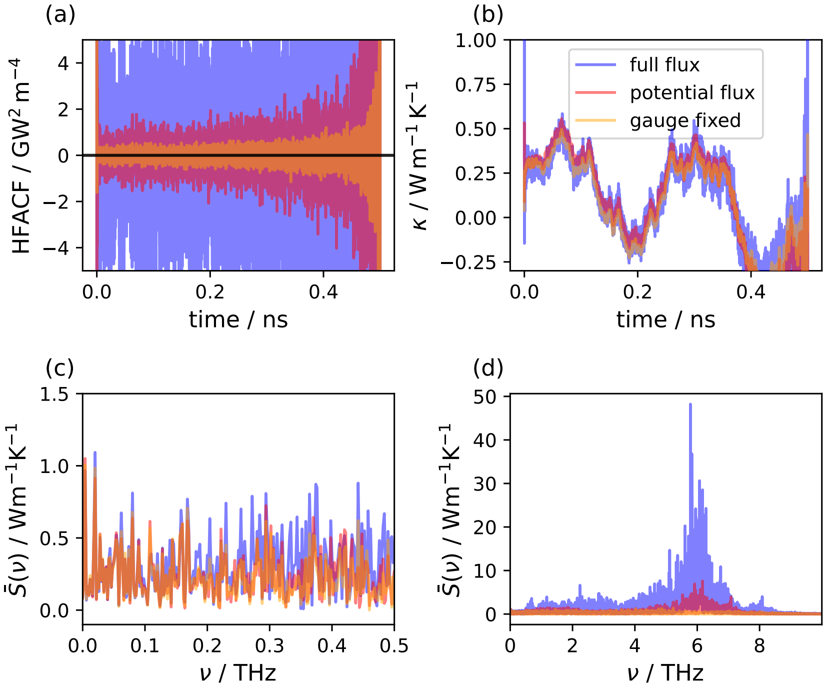

Figure S9: Comparison of different noise reduction techniques for the independent simulation number 6 for the ZB nanowire in the same style as figure S4.

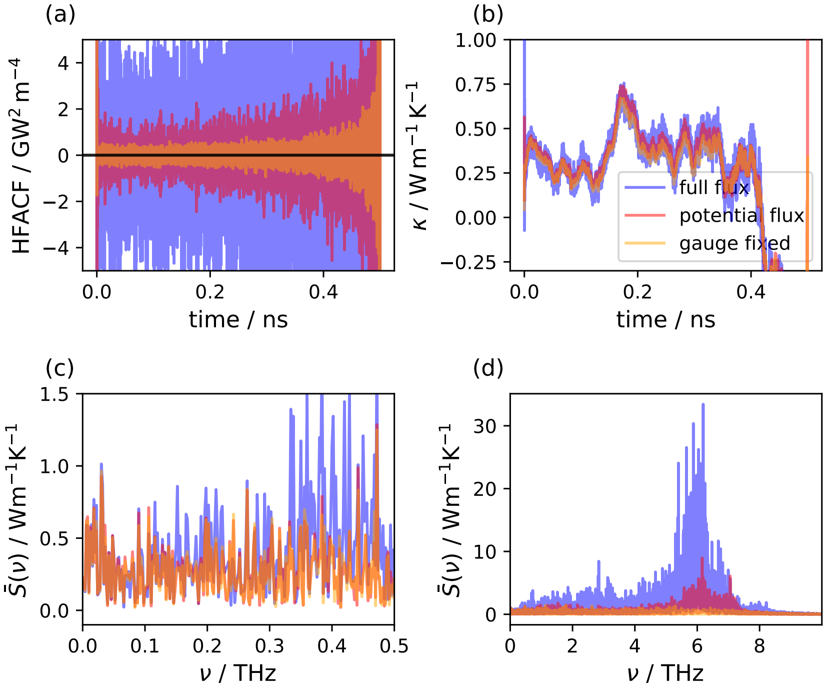

Figure S10: Comparison of different noise reduction techniques for the independent simulation number 7 for the ZB nanowire in the same style as figure S4.

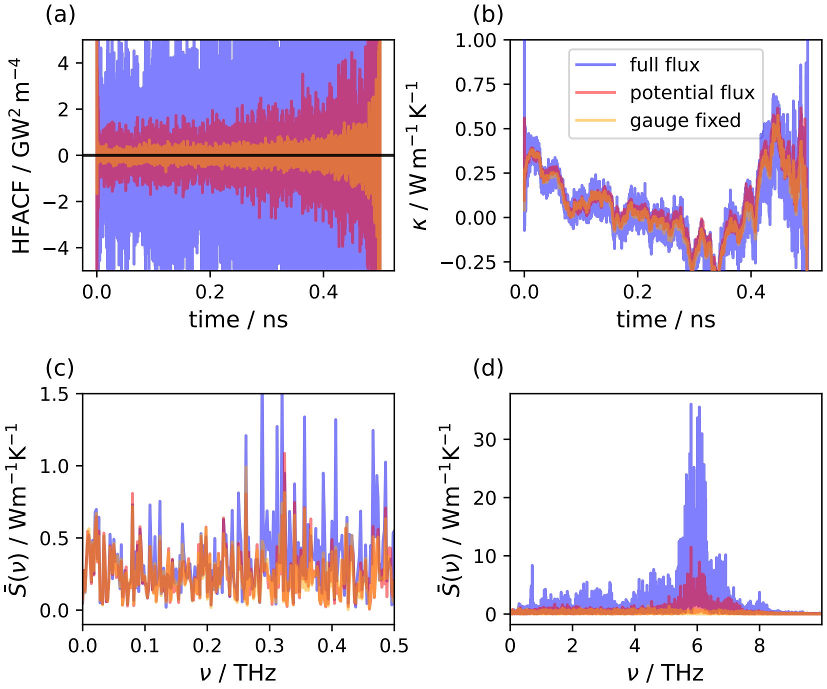

Figure S11: Comparison of different noise reduction techniques for the independent simulation number 8 for the ZB nanowire in the same style as figure S4.

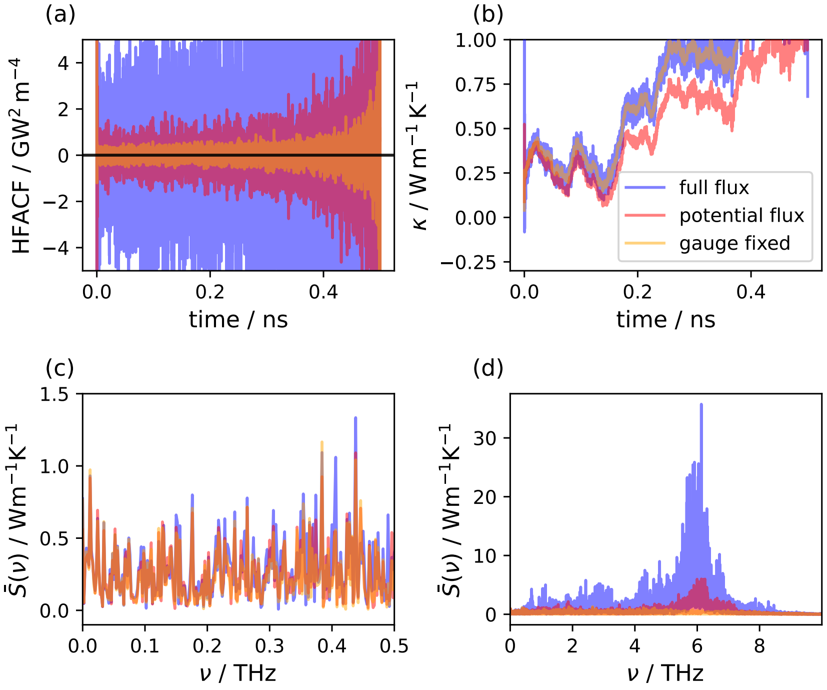

Figure S12: Comparison of different noise reduction techniques for the independent simulation number 9 for the ZB nanowire in the same style as figure S4.

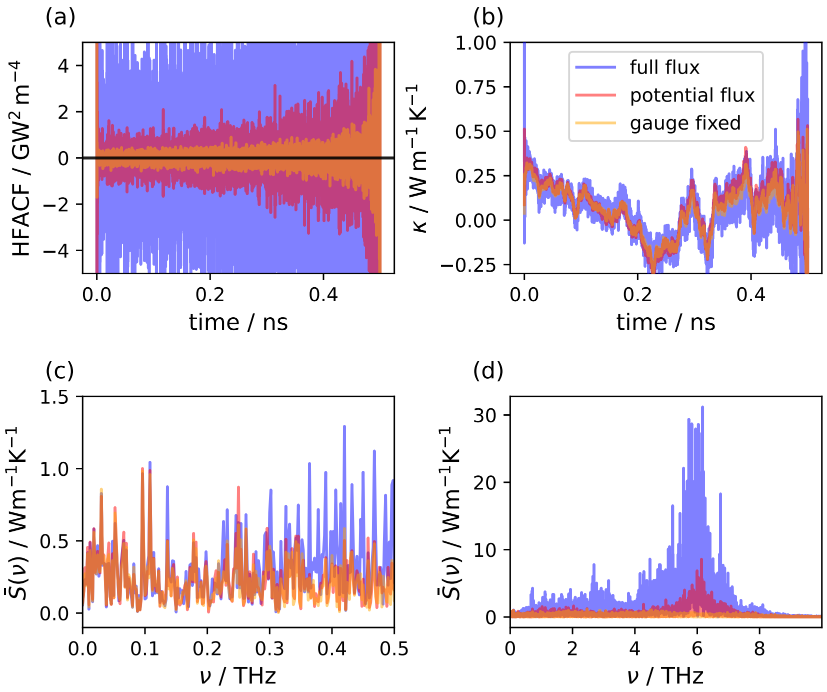

Figure S13: Comparison of different noise reduction techniques for the independent simulation number 10 for the ZB nanowire in the same style as figure S4.

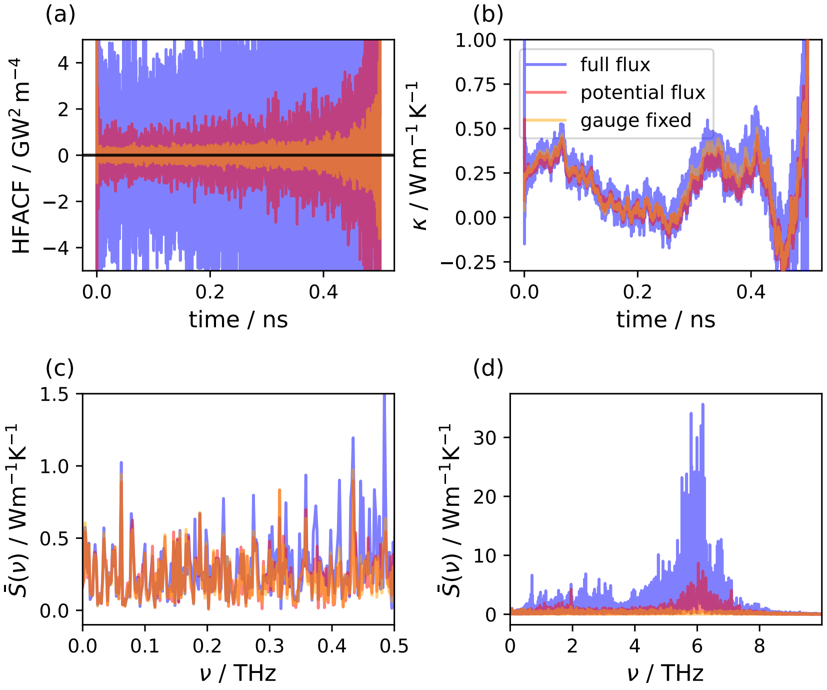

Figure S14: Comparison of different noise reduction techniques for the independent simulation number 11 for the ZB nanowire in the same style as figure S4.

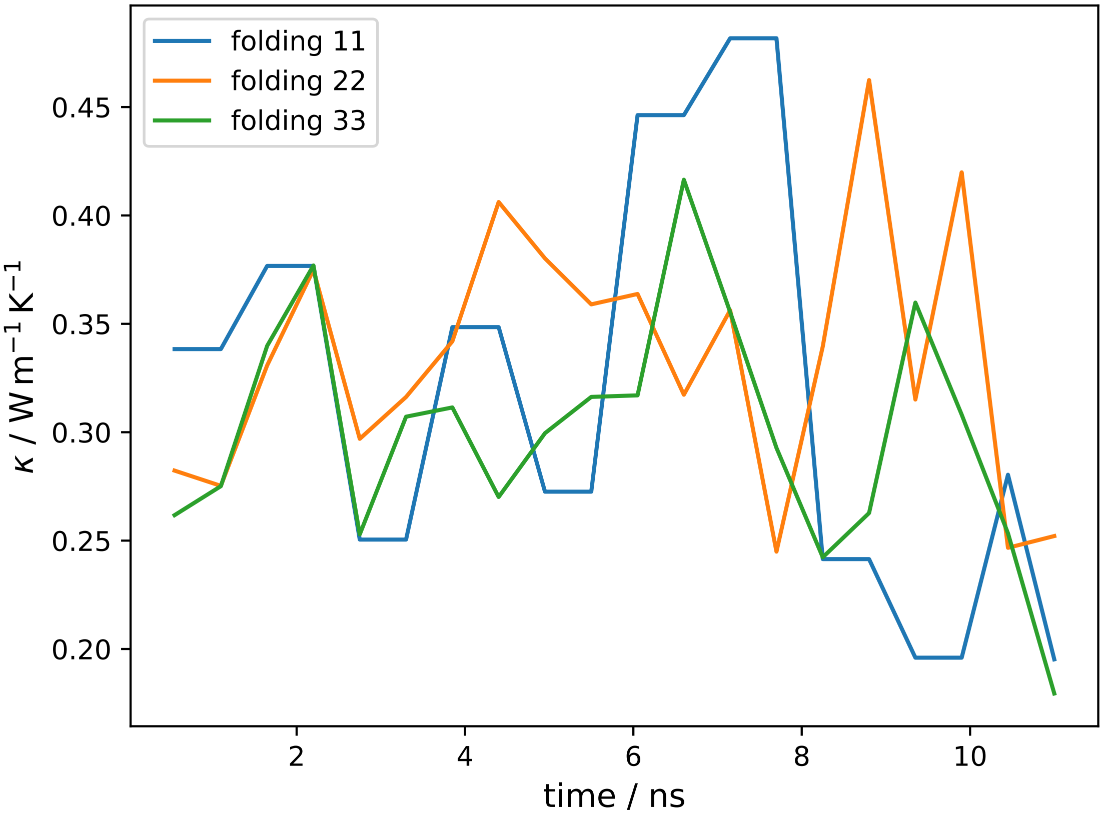

Figure S15: Figure showcasing the zero frequency thermal conductivity from the power spectrum for the ZB nanowire.

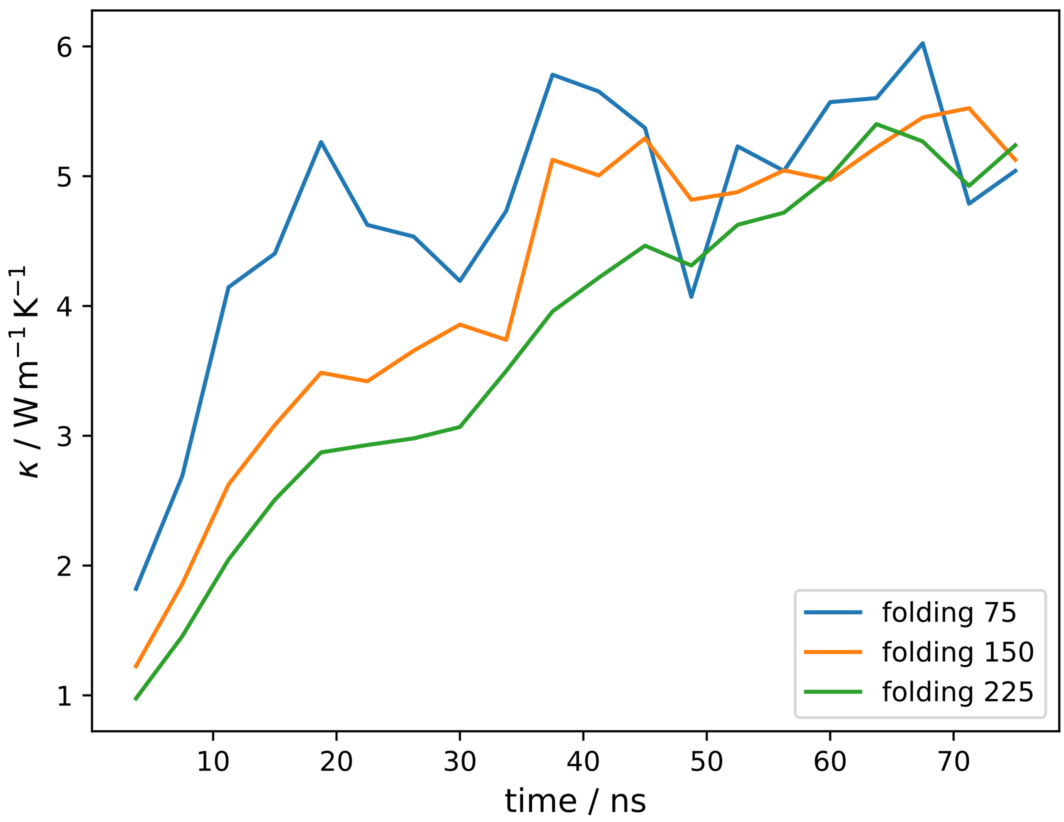

Figure S16: Figure showcasing the zero frequency thermal conductivity from the power spectrum for the WZ nanowire.

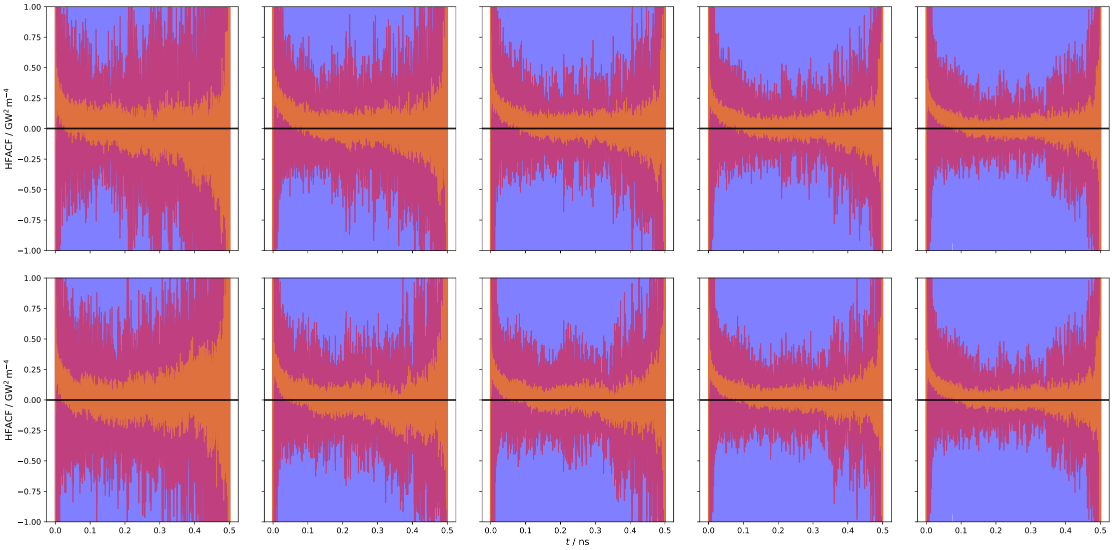

Figure S17: HFACFs depending on wether the number of independent simulations is increased (first row) or the total simulation time for each independent simulations (second row). The data is based on 5 5 ns long MD trajectories for the WZ nanowire. In the far left figures 5 ns of total simulation time is used while on the far right figure it accumulated to 25 ns

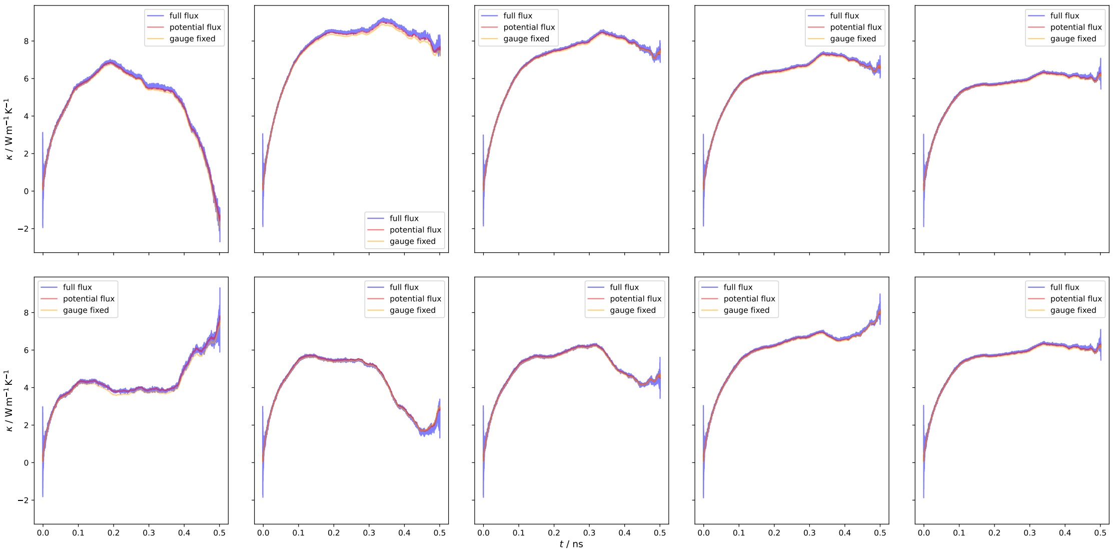

Figure S18: Thermal integral over the HFACFs depending on wether the number of independent simulations is increased (first row) or the total simulation time for each independent simulations (second row). The underlying data is the same as in Figure S17

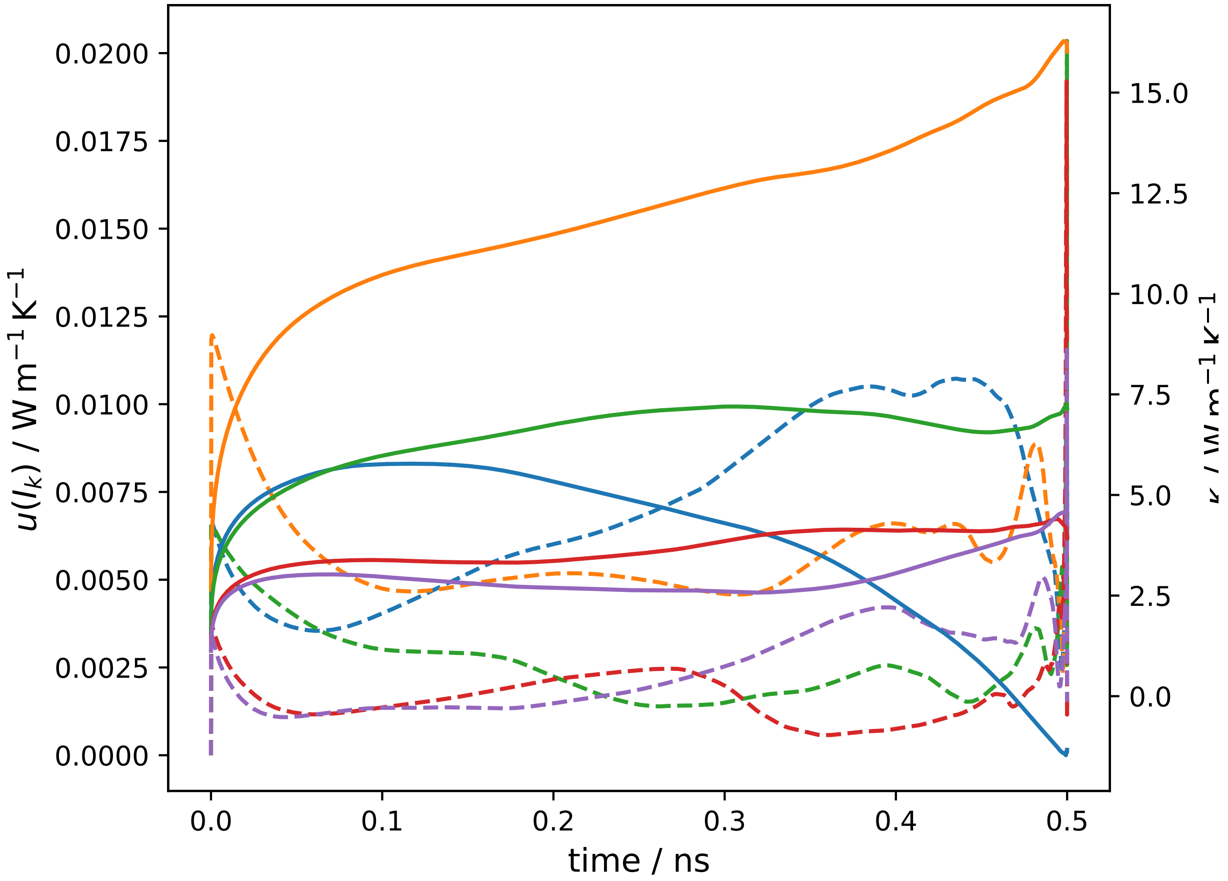

Figure S19: Thermal conductivities (solid lines) and uncertainties (dashed lines) from 5 individual 5 ns simulations for the WZ nanowire according to the prescribed KUTE approach after applying weighted averages to the individual runs.

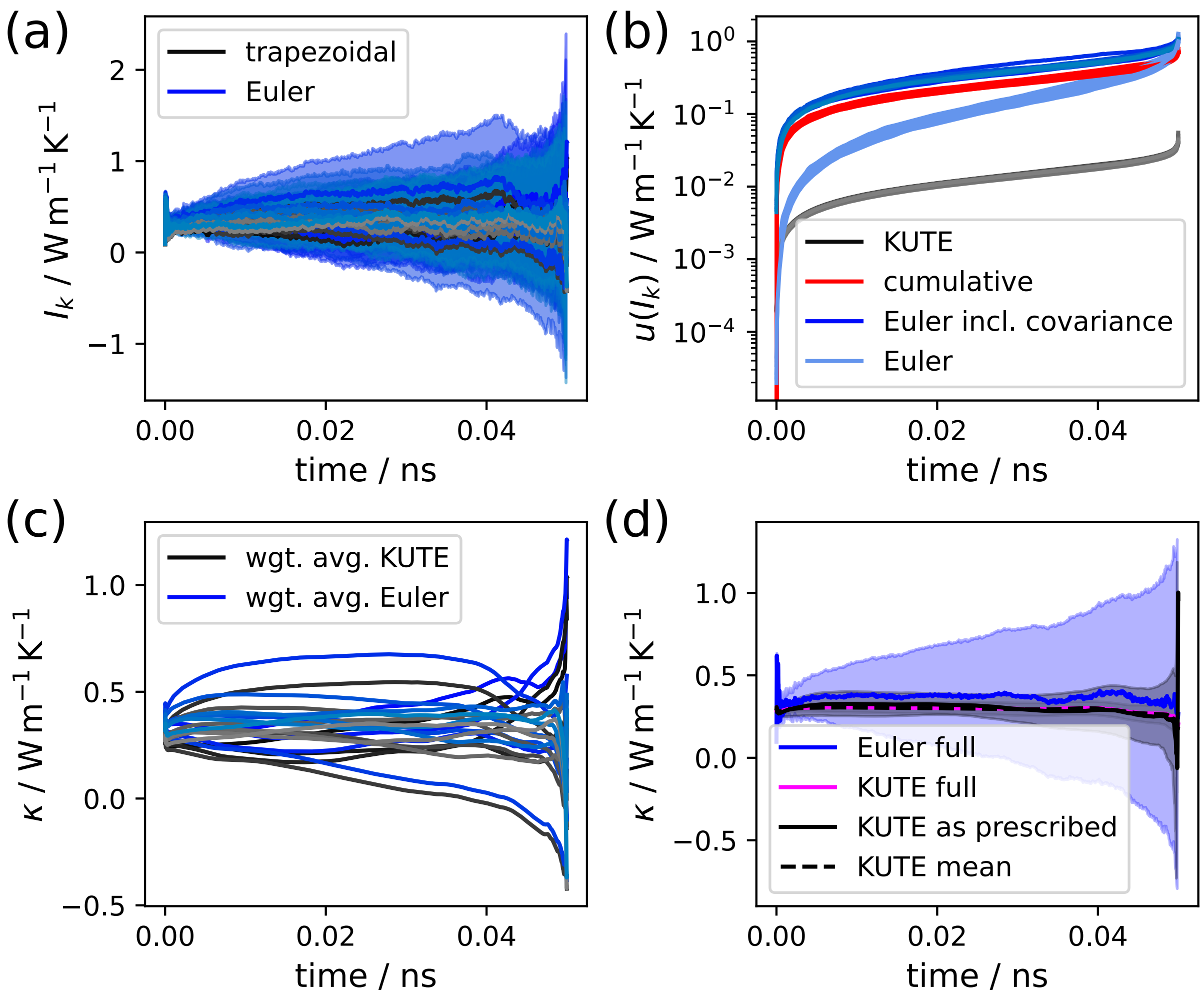

Figure S20: Comparison of analysis methods based on 11 1 ns GK simulations of the ZB nanowire. (a) Direct integration of a 20 times folded HFACF for the 11 independent runs using trapezoidal integration (black) or Euler integration (blue). The shaded area represents the uncertainty. (b) comparison of different uncertainty metrics. (c) Uncertainty weighted averages using the uncertainty including the covariance (blue) or the uncertainty as prescribed in the KUTE approach (black). (d) Comparison of different analysis techniques of the thermal conductivity with their uncertainty prediction. For a detailed description, see the main manuscript.

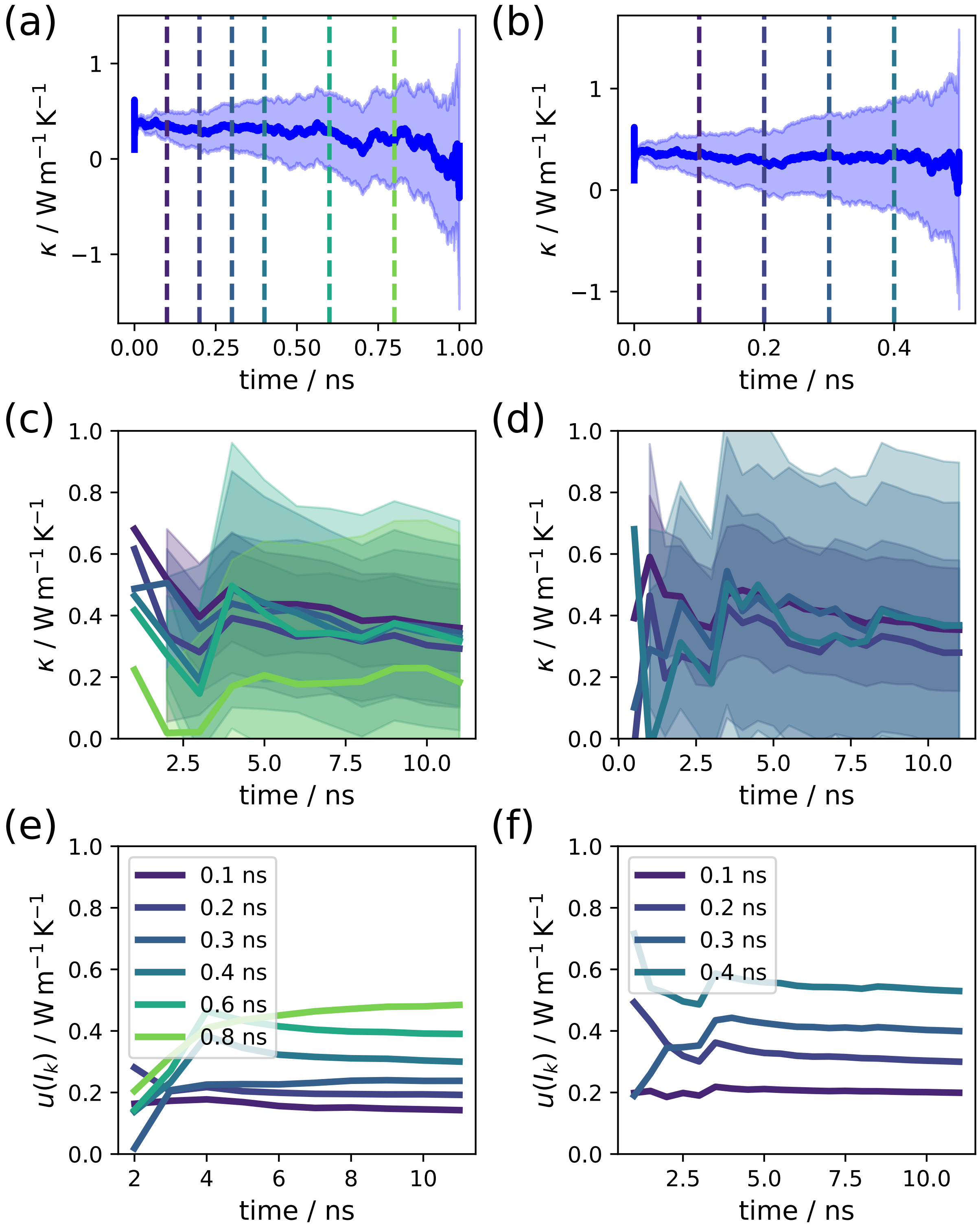

Figure S21: Time convergence of the ZB nanowire with a correlation time of 1 ns (a, c, e) or 0.5 ns (b, d, f) evaluated over a total simulation time of 11 ns. (a, b) The cumulative integrals performed using the Euler method and the corresponding uncertainty for the entire duration. (c, d) The time convergence curves based on values extracted after specific correlation times as indicated in (a,b). The corresponding uncertainties are shown in (e,f).
